# Correlates of COVID-19 vaccine hesitancy in Austria: trust and the government

**DOI:** 10.1093/pubmed/fdab122

**Published:** 2021-05-05

**Authors:** Eva Schernhammer, Jakob Weitzer, Manfred D Laubichler, Brenda M Birmann, Martin Bertau, Lukas Zenk, Guido Caniglia, Carlo C Jäger, Gerald Steiner

**Affiliations:** Department of Epidemiology, Center for Public Health, Medical University of Vienna, 1090 Vienna, Austria; Complexity Science Hub, 1080 Vienna, Austria; Channing Division of Network Medicine, Department of Medicine, Brigham and Women’s Hospital and Harvard Medical School, Boston, MA 02115, USA; Department of Epidemiology, Center for Public Health, Medical University of Vienna, 1090 Vienna, Austria; Complexity Science Hub, 1080 Vienna, Austria; School of Complex Adaptive Systems, Arizona State University, Tempe, AZ 85287, USA; Santa Fe Institute, Santa Fe, NM 87501, USA; Channing Division of Network Medicine, Department of Medicine, Brigham and Women’s Hospital and Harvard Medical School, Boston, MA 02115, USA; Institute for Technical Chemistry, TU Bergakademie Freiberg, 09599 Freiberg, Germany; Department for Knowledge and Communication Management, Danube University Krems, 3500 Krems and der Donau, Austria; Konrado Lorenz Institute for Evolution and Cognition Research, 3400 Klosterneuburg, Austria; Global Climate Forum, 10178 Berlin, Germany; Complexity Science Hub, 1080 Vienna, Austria; Institute for Technical Chemistry, TU Bergakademie Freiberg, 09599 Freiberg, Germany

**Keywords:** behaviour, communicable diseases, vaccine hesitancy, COVID-19

## Abstract

**Background:**

With the coronavirus disease 2019 (COVID-19) pandemic surging and new mutations evolving, trust in vaccines is essential.

**Methods:**

We explored correlates of vaccine hesitancy, considering political believes and psychosocial concepts, conducting a non-probability quota-sampled online survey with 1007 Austrians.

**Results:**

We identified several important correlates of vaccine hesitancy, ranging from demographics to complex factors such as voting behavior or trust in the government. Among those with hesitancy towards a COVID-19 vaccine, having voted for opposition parties (opp) or not voted (novote) were (95% Confidence Intervall (CI)opp, 1.44–2.95) to 2.25-times (95%CI_novote_, 1.53–3.30) that of having voted for governing parties. Only 46.2% trusted the Austrian government to provide safe vaccines, and 80.7% requested independent scientific evaluations regarding vaccine safety to increase willingness to vaccine.

**Conclusions:**

Contrary to expected, psychosocial dimensions were only weakly correlated with vaccine hesitancy. However, the strong correlation between distrust in the vaccine and distrust in authorities suggests a common cause of disengagement from public discourse.

## Introduction

Austria was among the first countries in Europe to report coronavirus disease 2019 (COVID-19) cases at the beginning of the pandemic.^1^ In response to the first and second wave of COVID-19, complete lockdown measures were implemented in Austria for 6 weeks between March 16 and 25 April 2020; and then again—with some interruption during the Christmas holidays in December—from 3 November 2020 through 8 February 2021.^2^

As the COVID-19 pandemic continues to surge worldwide, recent approvals of COVID-19 vaccines raise hope for a light at the end of the long and dark tunnel. But COVID-19 vaccination programs can affect meaningful resolution only with sufficient participation rates to achieve herd immunity. While a global survey from June 2020 revealed promising levels of potential COVID-19 vaccine acceptance (55–89%),^3^ in September, only 51% of US adults indicated willingness to be vaccinated. Similarly, also in other countries, COVID-19 risk perception and vaccine intentions have fluctuated throughout the pandemic.^4–6^ Acceptance levels for restrictions and trust in authorities has diminished, fueled in part by political polarization^7^; and difficulties with the vaccine roll-out and hesitancy towards a COVID-19 vaccine has increased.

In order to better deal with the underlying complexity of pandemic threats, our aim was to extend on the study by Volpp *et al*.,^8^ providing additional data in support of their core request of rebuilding trust in the rigor of vaccine trials and the integrity of the approval process. Specifically, we conducted a non-probability online survey, which was conducted during the period of the second lockdown and quota sampled to match the population in Austria for age, gender, and region. We explored attitudes and reasons for behavior towards governmentally mandated lockdown measures and correlates of vaccine hesitancy, including political views, voting behavior and also considering psychosocial concepts such as optimism, altruism, resilience, and need for cognitive closure, as well as altruistic reasons for behavior.

## Results

The median age of the whole sample was 42 years (interquartile range = 32–52). Of all 1007 survey respondents (498 men, 509 women), 414 (41.1%) reported intermediate or severe vaccine hesitancy [women: 236 (46.4%); men 178 (35.7%)], 230 men and women (22.8%) were undecided and 363 (36.1%) reported no or little hesitancy towards receiving a COVID-19 vaccine once available. Adherence to governmentally implemented COVID-19 measures partially or total was high (91.1%). Overall, vaccine hesitancy (intermediate or high) was more frequent among those aged 35–54 (51.1% versus 7.7% for those ages 60+), living in rural areas [towns or cities with <50.000 inhabitants; 54.8% versus 41.1% for those living in urban areas)], with a high school education or less (46.1% versus 33.6% with education levels higher than high school). Among participants who reported having voted for the political parties currently in power in Austria, 55.4% had little or no hesitancy towards a COVID-19 vaccine, compared to 26.7% (having voted for the opposition parties) and 17.9% among those who did not vote during the last national election. Further, vaccine acceptance was higher among those with high optimism (30.9%) compared to those with low optimism (19.4%), whereas vaccine hesitancy was high among those with high self-reported resilience (32.1%) versus those with low resilience (22.2%). Among those with least vaccine hesitancy, the least frequently used main sources of information about the pandemic were social media (14.6%) and friend/kin (9.6%), whereas in particular among those who were undecided regarding vaccine, social media were more frequently used as main source of information (21.7%). The most frequent reasons overall that were listed by participants why they did or did not follow the imposed Corona guidelines were ‘to protect own health’ (60.3%) and ‘to protect my family’s health’ (55.3%). Among those with little hesitancy towards a COVID-19 vaccine, 59.8% stated that they followed the Corona measures because they approved them; whereas among those with high hesitancy, 56.6% stated that they did not follow imposed measures because they did not approve them (Table 1). The question whether participants had trust in the Austrian government to provide a safe COVID-19 vaccine was answered with ‘yes, definitely’ by 10% of the overall sample, and 26.8% answered ‘definitely not’ (27% said ‘rather not’, and 36.2% ‘rather yes’). By contrast, 80.7% reported that an independent scientific evaluation of vaccine safety would (‘yes, definitely’, or ‘rather yes’) increase their willingness to get vaccinated.

**Table 1:** Sample characteristics across vaccine hesitancy in a sample (*N* = 1007) of the Austrian adult population

*Characteristics*	*Vaccine hesitancy*		
	*No or little hesitancy (*n* = 363)*	*Undecided (*n* = 230)*	*Intermediate or high hesitancy (*n* = 414)*
	**n* (%)*	**n* (%)*	**n* (%)*
**Gender**			
Men	203 (55.9)	117 (50.9)	178 (43.0)
**Age**			
<35 years	106 (29.2)	91 (39.6)	124 (30.0)
35–54 years	147 (40.5)	105 (45.6)	224 (54.1)
55–59 years	53 (14.6)	20 (8.7)	34 (8.2)
≥60 years	57 (15.7)	14 (6.1)	32 (7.7)
**Region of residence**			
Burgenland	13 (3.6)	8 (3.5)	17 (4.1)
Carinthia	22 (6.1)	10 (4.4)	28 (6.7)
Lower Austria	66 (18.2)	49 (21.3)	96 (23.2)
Salzburg	17 (4.7)	15 (6.5)	30 (7.3)
Styria	53 (14.6)	27 (11.7)	57 (13.8)
Tyrol	21 (5.8)	18 (7.8)	28 (6.8)
Upper Austria	51 (14.1)	26 (11.3)	54 (13.0)
Vienna	113 (31.1)	70 (30.4)	79 (19.1)
Vorarlberg	7 (1.9)	7 (3.0)	25 (6.0)
**Area of residence**			
Urban	197 (54.3)	105 (45.6)	170 (41.1)
Rural	166 (45.7)	125 (54.4)	244 (58.9)
**Education**			
High school or less	123 (33.9)	86 (37.4)	191 (46.1)
Matura (university entry exam)	148 (40.8)	96 (41.7)	139 (33.6)
University degree	92 (25.3)	48 (20.9)	84 (20.3)
Child, <16 years	85 (23.4)	75 (32.6)	118 (28.5)
**Political party preference**			
Governing	201 (55.4)	124 (53.9)	154 (37.2)
Opposition	97 (26.7)	54 (23.5)	135 (32.6)
Did not vote (last elections)	65 (17.9)	52 (22.6)	125 (30.2)
**Optimism [quartiles]**			
Low	71 (19.4)	27 (11.7)	90 (21.7)
Low-intermediate	83 (22.9)	83 (36.1)	117 (28.3)
Intermediate-high	97 (26.8)	61 (26.5)	94 (22.7)
High	112 (30.9)	59 (25.7)	113 (27.3)
**Resilience [quartiles]**			
Low	80 (22.0)	73 (31.8)	92 (22.2)
Low-intermediate	99 (27.3)	58 (25.2)	101 (24.4)
Intermediate-high	86 (23.7)	50 (21.7)	88 (21.3)
High	98 (27.0)	49 (21.3)	133 (32.1)
**Need for cognitive closure [quartiles]**			
Low	94 (25.9)	58 (25.2)	91 (21.9)
Low-intermediate	82 (22.6)	72 (31.3)	103 (24.9)
Intermediate-high	83 (22.9)	44 (19.1)	98 (23.7)
High	104 (28.7)	56 (24.4)	122 (29.5)
**Main source to inform oneself about measures/recommendations**			
TV	216 (59.5)	100 (43.5)	226 (54.6)
Newspaper (paper format)	76 (20.9)	35 (15.2)	70 (16.9)
Newspaper (online format)	102 (28.1)	54 (23.5)	99 (23.9)
Radio	69 (19.0)	37 (16.1)	69 (16.7)
Official web pages of the Austrian ministries	86 (23.7)	61 (26.5)	62 (15.0)
Social media	53 (14.6)	50 (21.7)	99 (23.9)
Friends/kin	35 (9.6)	27 (11.7)	44 (10.6)
**Frequency of informing oneself**			
More than once a day	131 (36.1)	66 (28.7)	91 (22.0)
Once a day	147 (40.5)	84 (36.5)	164 (39.6)
Several times a week	68 (18.7)	55 (23.9)	87 (21.0)
Weekly or less often	17 (4.7)	25 (10.9)	72 (17.4)
**Perceived risk of infection between June–October 2020**			
Negligible	38 (10.5)	20 (8.7)	100 (24.2)
Low	156 (42.9)	116 (50.4)	186 (44.9)
Intermediate	124 (34.2)	73 (31.7)	94 (22.7)
High	45 (12.4)	21 (9.1)	34 (8.2)
**Subjective health status**			
Very good	97 (26.7)	55 (23.9)	128 (30.9)
Good	185 (51.0)	116 (50.4)	175 (42.3)
Fair, bad or very bad	81 (22.3)	59 (25.7)	111 (26.8)
**Overall adherence to measures**			
Yes, to protect my health	279 (79.5)	146 (68.5)	182 (50.4)
Yes, do want to quarantine	112 (31.9)	62 (29.1)	95 (26.3)
Yes, do approve measures	210 (59.8)	105 (49.3)	114 (31.6)
Yes, to protect my family’s health	247 (70.4)	133 (62.4)	177 (49.0)
Yes, to protect the population’s health	192 (54.7)	90 (42.3)	101 (28.0)
Yes, the government said so	92 (26.2)	46 (21.6)	112 (31.0)
Yes, do not want to cause death	136 (38.8)	68 (31.9)	72 (19.9)
No, COVID-19 is no danger	4 (33.3)	5 (29.4)	20 (37.7)
No, did not approve measures	6 (50.0)	8 (47.1)	30 (56.6)
No, did not understand measures	3 (25.0)	3 (17.7)	9 (17.0)
No, burden for economy	1 (8.3)	5 (29.4)	11 (20.8)
No, social burden	3 (25.0)	3 (17.8)	21 (39.6)
**Change in quality of life (compared to before COVID)**			
Decrease	109 (30.0)	74 (32.2)	129 (31.2)
No change	212 (58.4)	125 (54.3)	239 (57.7)
Increase	42 (11.6)	31 (13.5)	46 (11.1)

Next, we examined associations between correlates of vaccine hesitancy taking into account potential confounders. Because results from age- and multivariable models were largely similar, we focused on the multivariable results (Table 2). Men were significantly less likely than women to report (intermediate or high) vaccine hesitancy (odds ratio (OR) = 0.56; 95%CI, 0.41–0.76). Compared to those aged 35 or younger, vaccine hesitancy appeared to decline with increasing age (OR_35-54yrs_ = 1.20, 95%CI, 0.83–1.73; OR_55-59yrs_ = 0.48, 95%CI, 0.28–0.84; and OR_60 + yrs_ = 0.37, 95%CI, 0.21–0.66). Further, compared to those who live in urban areas, those in smaller rural areas had a significantly higher odds of intermediate to high vaccine hesitancy (OR = 1.86, 95%CI, 1.36–2.54). The highest odds of expressing vaccine hesitancy was reported by those who voted for opposition parties (OR = 2.06; 95%CI, 1.44–2.95) and those who did not vote during the last national election (OR = 2.25; 95%CI, 1.53–3.30). Individuals with intermediate to high optimism were significantly less likely to express vaccine hesitancy (OR = 0.71; 95%CI, 0.51–0.99). Resilience, need for cognitive closure, preferred main source of information regarding Corona, and subjective health status did not correlate significantly with vaccine hesitancy.

**Table 2:** Correlates of vaccine hesitancy in a sample (*N* = 1007) of the Austrian adult population

	*Vaccine hesitancy*				
	*No or little hesitancy (*N* = 363)*	*Undecided (*N* = 230)*		*Intermediate or high hesitancy (*N* = 414)*	
	*N* (%)	*N* (%)	OR (95% CI)^*^	*N* (%)	OR (95% CI)^*^
**Gender**					
Men	230 (55.9)	117 (50.9)	0.80 (0.57–1.13)	178 (43.0)	0.56 (0.41–0.76)
**Age**					
<35 year	106 (29.2)	91 (39.6)	1	124 (30.0)	1
35–54 year	147 (40.5)	105 (45.6)	0.82 (0.54–1.23)	224 (54.1)	1.20 (0.83–1.73)
55–59 year	53 (14.6)	20 (8.7)	0.40 (0.22–0.76)	34 (8.2)	0.48 (0.28–0.84)
≥60 year	57 (15.7)	14 (6.1)	0.27 (0.14–0.54)	32 (7.7)	0.37 (0.21–0.66)
**Area of residence**					
Urban	197 (54.3)	105 (45.6)	1	170 (41.1)	1
Rural ≥ 50 K	20 (5.5)	14 (6.1)	1.36 (0.65–2.85)	17 (4.1)	0.98 (0.48–1.99)
Rural < 50 K	146 (40.2)	111 (48.3)	1.45 (1.02–2.07)	227 (54.8)	1.86 (1.36–2.54)
**Education**					
Middle school or less	123 (33.9)	86 (37.4)	1	191 (46.1)	1
High school	148 (40.8)	96 (41.7)	0.82 (0.55–1.22)	139 (33.6)	0.64 (0.45–0.91)
University degree	92 (25.3)	48 (20.9)	0.66 (0.41–1.05)	84 (20.3)	0.58 (0.39–0.88)
**Political party preference**					
Governing	201 (55.4)	124 (53.9)	1	154 (37.2)	1
Opposition	97 (26.7)	54 (23.5)	1.00 (0.66–1.52)	135 (32.6)	2.06 (1.44–2.95)
Did not vote	65 (17.9)	52 (22.6)	1.16 (0.74–1.80)	125 (30.2)	2.25 (1.53–3.30)
**Optimism**					
Low/low-intermed	154 (42.4)	110 (47.8)	1	207 (50.0)	1
Intermed-high/high	209 (57.6)	120 (52.2)	0.98 (0.67–1.43)	207 (50.0)	0.71 (0.51–0.99)
**Resilience**					
Low/low-intermed	179 (49.3)	131 (57.0)	1	193 (46.6)	1
Intermed-high/high	184 (50.7)	99 (43.0)	0.91 (0.63–1.33)	221 (53.4)	1.37 (0.98–1.92)
**Need for cognitive closure**					
Low/low-intermed	176 (48.5)	130 (56.5)	1	194 (46.9)	1
Intermed-high/high	187 (51.5)	100 (43.5)	0.71 (0.50–0.99)	220 (53.1)	1.01 (0.74–1.36)
**Main source to inform oneself about measures/recommendations**					
TV	216 (59.5)	100 (43.5)	1.03 (0.72–1.48)	226 (54.6)	0.88 (0.64–1.20)
**Subjective health status**					
Very good	97 (26.7)	55 (23.9)	1	128 (30.9)	1
Good	185 (51.0)	116 (50.4)	1.05 (0.69–1.60)	175 (42.3)	0.70 (0.49–1.01)
Fair, bad or very bad	81 (22.3)	59 (25.7)	1.23 (0.73–2.05)	111 (26.8)	0.98 (0.63–1.53)

^*^Risk estimates are ORs with 95% CIs adjusted for all variables presented in the table.

**Table 3:** Correlates of vaccine hesitancy among participants (*N* = 654) who indicated overall adherence to COVID-19 measures and reported an ‘altruistic’ reason^#^ for their adherence

	*Vaccine hesitance*				
	*No or little hesitancy (*N* = 286)*	*Undecided (*N* = 156)*		*Intermediate or high hesitancy (*N* = 212)*	
	*N* (%)	*N* (%)	OR (95% CI)^*^	*N* (%)	OR (95% CI)^*^
**Gender**					
Men	154 (53.9)	71 (45.5)	0.74 (0.49–1.11)	71 (33.5)	0.42 (0.28–0.63)
**Age**					
<35 years	82 (28.6)	61 (39.1)	1	66 (31.1)	1
35–54 years	120 (42.0)	72 (46.1)	0.68 (0.42–1.10)	113 (53.3)	1.01 (0.64–1.61)
55–59 years	40 (14.0)	16 (10.3)	0.39 (0.19–0.82)	23 (10.9)	0.56 (0.28–1.10)
≥60 years	44 (15.4)	7 (4.5)	0.17 (0.07–0.42)	10 (4.7)	0.21 (0.09–0.50)
**Area of residenc**					
Urban	168 (58.7)	67 (43.0)	1	82 (38.7)	1
Rural ≥ 50.000 inhabitants	11 (3.9)	9 (5.7)	2.09 (0.80–5.46)	9 (4.2)	1.50 (0.56–4.01)
Rural < 50.000 inhabitants	107 (37.4)	80 (51.3)	1.93 (1.27–2.95)	121 (57.1)	2.27 (1.52–3.38)
**Education**					
High school or less	93 (32.5)	58 (37.2)	1	98 (46.2)	1
Matura (university entry exam)	114 (39.9)	61 (39.1)	0.77 (0.47–1.25)	72 (34.0)	0.65 (0.41–1.03)
University degree	79 (27.6)	37 (23.7)	0.66 (0.38–1.15)	42 (19.8)	0.49 (0.29–0.82)
**Political party preference**					
Governing	157 (54.9)	87 (55.8)	1	88 (41.5)	1
Opposition	80 (28.0)	34 (21.8)	0.88 (0.53–1.77)	56 (26.4)	1.53 (0.96–2.46)
Did not vote (last elections)	49 (17.1)	35 (22.4)	1.13 (0.67–1.92)	68 (32.1)	2.16 (1.33–3.51)
**Optimism**					
Low or low-intermediate	114 (39.9)	63 (40.4)	1	100 (47.2)	1
Intermediate-high or high	172 (60.1)	93 (59.6)	1.13 (0.72–1.77)	112 (52.8)	0.77 (0.50–1.19)
**Resilience**					
Low or low-intermediate	137 (47.9)	74 (47.4)	1	94 (44.3)	1
Intermediate-high or high	149 (52.1)	82 (52.6)	1.24 (0.80–1.93)	118 (55.7)	1.49 (0.97–2.28)
**Need for cognitive closure**					
Low or low-intermediate	144 (50.4)	82 (52.6)	1	100 (47.2)	1
Intermediate-high or high	142 (49.6)	74 (47.4)	0.86 (0.57–1.30)	112 (52.8)	0.95 (0.64–1.40)
**Main source to inform oneself about measures/recommendations**					
TV	114 (39.9)	62 (39.7)	1.20 (0.79–1.83)	88 (41.5)	1.11 (0.75–1.66)
**Subjective health status**					
Very good	76 (26.6)	39 (25.0)	1	63 (29.7)	1
Good	148 (51.7)	83 (53.2)	1.08 (0.66–1.78)	90 (42.5)	0.70 (0.44–1.11)
Fair, bad or very bad	62 (21.7)	34 (21.8)	1.15 (0.61–2.15)	59 (27.8)	1.02 (0.58–1.79)

^#^Protection of family’s health, protection of the population’s health, or not wanting to cause the death of another person.

^*^Risk estimates are ORs with 95% confidence intervals adjusted for all variables presented in the table.

In secondary analyses, we stratified our sample by grouping participants’ answers into altruistic reasons (Table 3) versus non-altruistic reasons (Table 4) for behaviour towards Corona measures, as described in the Methods section. Among the 654 participants who were categorized as reporting ‘altruistic reasons’ as primary drivers for their behaviour during the pandemic [358 women (54.7%) and 296 men (45.2%)], correlations between covariables and vaccine hesitancy remained similar to overall though they were slightly more pronounced for men, higher age (60+), and small rural areas (Table 3), suggesting that these groups were least hesitant towards a COVID-19 vaccine. For the group who did not report any of the three selected altruistic reasons (*n* = 271, 119 women (43.9%) and 152 men (56.1%), Table 4), the most striking differences among those with hesitancy towards a COVID-19 vaccine were that the odds of having higher education appeared less pronounced (OR = 0.81; 95%CI, 0.33–1.99), compared to the total sample; and that the odds of having voted for opposition parties was 3.5-times that of those who voted for the governing parties. Overall, trust in the Austrian government to provide a safe vaccine was low (53.8% indicated rather or definitely not trust), whereas the desire for independent scientific evaluations regarding vaccine safety to increase willingness to vaccine was high (80.7%).

**Table 4:** Correlates of vaccine hesitancy among participants (*N* = 271) who indicated overall adherence to COVID-19 measures and did not report an ‘altruistic’ reason^#^ for their adherence

	*Vaccine hesitancy*				
	*No or little hesitancy (*N* = 65)*	*Undecided (*N* = 57)*		*Intermediate or high hesitancy (*N* = 149)*	
	*N* (%)	*N* (%)	OR (95% CI)^*^	*N* (%)	OR (95% CI)^*^
**Gender**					
Men	42 (64.6)	34 (59.7)	0.66 (0.29–1.48)	75 (50.3)	0.50 (0.24–0.92)
**Age**					
<35 years	17 (26.1)	23 (40.3)	1	38 (25.5)	1
35–54 years	23 (35.4)	24 (42.1)	0.89 (0.34–2.32)	81 (54.4)	1.48 (0.64–3.42)
55–59 years	12 (18.5)	3 (5.3)	0.21 (0.05–0.99)	10 (6.7)	0.31 (0.10–1.01)
≥60 years	13 (20.0)	7 (12.3)	0.46 (0.13–1.62)	20 (13.4)	0.46 (0.16–1.29)
**Area of residence**					
Urban	23 (35.4)	27 (47.4)	1	70 (46.9)	1
Rural ≥ 50.000 inhabitants	8 (12.3)	5 (8.7)	0.53 (0.13–2.10)	5 (3.4)	0.16 (0.04–0.61)
Rural < 50.000 inhabitants	34 (52.3)	25 (43.9)	0.59 (0.26–1.35)	74 (49.7)	0.77 (0.39–1.50)
**Education**					
High school or less	25 (38.5)	21 (36.8)	1	71 (47.7)	1
Matura (university entry exam)	27 (41.5)	27 (47.4)	0.97 (0.40–2.38)	47 (31.5)	0.57 (0.27–1.20)
University degree	13 (20.0)	9 (15.8)	0.74 (0.24–2.29)	31 (20.8)	0.81 (0.33–1.99)
**Political party preference**					
Governing	37 (57.0)	29 (50.9)	1	52 (34.9)	1
Opposition	14 (21.5)	16 (28.1)	1.65 (0.64–4.22)	56 (37.6)	3.58 (1.61–7.94)
Did not vote (last elections)	14 (21.5)	12 (21.0)	0.79 (0.28–2.17)	41 (27.5)	1.82 (0.81–4.12)
**Optimism**					
Low or low-intermediate	31 (47.7)	35 (61.4)	1	79 (53.0)	1
Intermediate-high or high	34 (52.3)	22 (38.6)	0.82 (0.34–1.99)	70 (47.0)	0.65 (0.31–1.38)
**Resilience**					
Low or low-intermediate	32 (49.2)	43 (75.4)	1	71 (47.7)	1
Intermediate-high or high	33 (50.8)	14 (24.6)	0.36 (0.14–0.90)	78 (52.3)	1.19 (0.58–2.45)
Need for cognitive closure					
Low or low-intermediate	23 (35.4)	34 (59.7)	1	63 (42.3)	1
Intermediate-high or high	42 (64.6)	23 (40.4)	0.40 (0.18–0.89)	86 (57.7)	0.86 (0.44–1.68)
**Main source to inform oneself about measures/recommendations**					
TV	40 (61.5)	30 (52.6)	0.88 (0.39–1.99)	77 (51.9)	0.61 (0.31–1.21)
**Subjective health status**					
Very good	20 (30.8)	16 (28.1)	1	49 (32.9)	1
Good	28 (43.1)	27 (47.4)	1.09 (0.42–2.81)	62 (41.6)	1.10 (0.50–2.42)
Fair, bad or very bad	17 (26.1)	14 (24.5)	0.76 (0.25–2.33)	38 (25.5)	0.90 (0.36–2.23)

^#^Protection of family’s health, protection of the population’s health, or not wanting to cause the death of another person.

^*^Risk estimates are ORs with 95% CI adjusted for all variables presented in the table.

Because voting behavior was the strongest correlate of vaccine hesitancy in our sample, we examined characteristics of the survey respondents stratified by voting behavior (voted for parties in power; voted for opposing parties; did not vote). We observed that the characteristics of those who did not vote during the last national election resembled those of persons with intermediate to high vaccine hesitancy: non-voters tended to be female, younger (<35 years), were more likely from the Austrian federal states Styria or Tyrol, more likely to be less educated (high school or less), they were substantially more likely to have vaccine hesitancy, less optimistic and to use as main source of information about the Corona pandemic their friends or social media, and to do so less frequently, compared to those who voted (regardless whether they voted for the parties in power or the opposition parties). In addition, they were more likely to perceive the risk of infecting themselves with COVID-19 as negligible or low, their subjective health status tended to be worse, and their life quality more likely to have changed for the worse during the pandemic, compared to Austrians who had voted during the last national election (Supplementary eTable 1).

## Discussion

### Main findings of this study

In our study, which reflects the main demographics of the Austrian population, 53.8% declared that they did not trust the Austrian government with respect to a safe vaccine, and merely 36.1% indicated that they would be likely to get vaccinated once a COVID-19 vaccine is available in Austria (with 22.8% undecided). In line with previous reports, the unwillingness to vaccinate was higher among women and younger Austrians^9^^,^^10^; but also among those in favor of political opposition parties (47.2%) or who did not vote in the last national election (51.6%) whereas it was lowest among survey respondents who indicated that they had voted for the parties in power (32.2%). By contrast, psychosocial concepts, with the exception of optimism, were not strongly correlated with willingness to vaccinate.

### What is already known on this topic

A survey from 2015, conducted among 350 patients visiting an emergency department in Austria has previously demonstrated high hesitancy towards vaccines with 50.3% having some reservations or no trust at all in vaccines, citing distrust in pharmaceutical industry, doubts regarding vaccines’ effectiveness, and fear of side effects.^11^ Physicians were the most trusted source of information regarding vaccines in that, as well as another small survey conducted in 2016 among a rural population (*n* = 306) in Austria.^12^ Clearly, against the backdrop of this general phenomenon of vaccine refusal in Austria, attitudes towards the COVID-19 vaccine warrant particular attention.

In our sample, vaccine hesitancy and voting behavior seem to converge. Reasons for rejecting Corona measures and protests against government interventions can have a wide variety of causes.^13^ The last Austrian national election, to which our question about voting behavior referred was held on 29 September 2019, i.e. long enough before the pandemic began. Of note, voter turnout had dropped to 75.6%,—the second-lowest turnout ever.^14^ In this context, our findings may be indicative of more profound societal changes and increasing distrust in the political system that can currently be observed not only in Austria but to some extent in all Western democracies,^15^ considering conspicuous phenomena such as democracy or state deniers (similar to the German ‘Querdenker’ or US ‘QAnon’ movements that deny the existence of the Coronavirus pandemic, among other conspiracy theories). In fact, vaccine hesitancy could be seen as a symptom of a much deeper problem, namely rejection of government or state action. These sentiments may be driven by an underlying sense of mistrust and fear, which has been proven to be an important factor, particularly in the context of the severe acute respiratory syndrome (SARS-CoV-2) pandemic.^16^

### What this study adds

Fear and uncertainty can be countered by improving the understanding of system complexity and the associated risks. In this context, the role and responsibilities of scientists must also be rethought. A current problem is the divergence of disciplinary perspectives even among scientists in the same discipline, which is proving counterproductive to building trust in proposed interventions or vaccines. These tendencies are exacerbated by scientists who become activists rather than following a rigorous scientific approach. Given the generally high level of distrust of government and the unfortunate politicization of much of the COVID-19 response, it is a call for scientists to become more assertive and communicate facts, gaps in current knowledge, and consequences for action independent of the political process. Because, among other things, the COVID-19 pandemic has shown that science can no longer be separated from society.

Mistrust and misinformation^17^ regarding a COVID-19 vaccine are widespread among the population worldwide, including in Austria. This could hinder pandemic response efforts and calls for a better understanding of those who exhibit particular vaccine hesitancy. Our findings of a strong correlation between vaccine hesitancy, unwillingness to follow Corona measures and political apathy or opposition are therefore relevant and troubling. They point to larger trends towards weakening societal cohesion and increasing distrust in institutions. Consistent with this, the Austrian Corona Panel Project^18^ found that satisfaction with government performance and the degree of solidarity in Austrian society had already declined from April to June 2020.

### Limitations of this study

Our study has several limitations of note, which include its cross-sectional nature and reliance on self-report for many covariables. Because the survey was conducted online, our reach of age groups beyond age 70 was limited due to their restricted use of digital media.

In conclusion, our results reflect a lack of trust in the political system,^15^^,^^19^ but indicate that there is possibility for improvement. In our survey, more than 80% wanted to see the results of an independent scientific evaluation of the vaccine before getting vaccinated. This supports—as previously suggested by Bauchner *et al*.^20^ and Cordero^21^—that an independent and de-politicized, rigorous, science-based evaluation of COVID-19 vaccines and transparent communication strategies^22^^,^^23^ can increase the willingness to be vaccinated. As part of the vaccine rollout, it will thus be essential to invest in transparent and effective ways to communicate results and achievements to the broader public. Our findings further imply that a diversified communication strategy is needed to address all citizens. Future studies should examine the extent of the relationship between trust in government and interventions, such as vaccination, in an international comparison to form the basis for meta-national communication and intervention strategies in dealing with pandemic threats.

## Methods

### Study population

Data collection was performed implementing a non-probability online survey between 26 November and 3 December 2020 among 1007 Austrian adults, quota sampled to match the population for age, gender and region. The survey was designed by members of the research team and implemented by the market research institute INTERROGARE, Bielefeld, Germany, using online panels to select participants who were representative of the Austrian population in terms of their residence, gender, and age. Because the survey was implemented online, only age groups up to around age 70 were reached (due to the still low digital literacy rate among higher age groups in Austria).

The survey was answered by 1007 adult participants aged 18 to 70 (498 male and 509 female), and comprised 60 questions on lifestyle, health and COVID-19 related measures and behaviour as well as attitudes toward a COVID-19 vaccine once available. It took on average 15 minutes to complete. Participants’ consent was implied by filling in the online survey. The study was exempt from Institutional Review Board approval according to Federal Regulations 45 CFR 46.10(b).

### Covariables

In addition to age, gender, lifestyle (smoking behaviour, body mass index, alcohol, caffeine consumption, diet, physical activity), health (previous SARS-CoV-2 infection; general health status, chronic disease history, use of supplements or medication, mental/emotional health) and sociodemographic variables (employment status, occupation, education, marital status, voting behaviour during last national elections, which took place in September 2019), several psychosocial attributes were also assessed, including *optimism* (using the validated Life Orientation Test LOT-revised^24^), *resilience* (using a German version^25^ of the resilience scale by Wagnild and Young,^26^ and *need for cognitive closure* (using the German short scale ‘need for cognitive closure’,^27^ validated according to the personality construct ‘need for cognitive closure’ (NCC) by Webster and Kruglanski^28^).

Regarding the pandemic and COVID-19, the survey elicited information on whether or not participants worked from home during the pandemic, use of media (and which type) to receive information about COVID, change in life quality since the beginning of the pandemic, what helped most to cope with COVID measures (predefined categories), and knowledge regarding, agreement with and acceptance of COVID measures that remained implemented by the Austrian government after the first wave/lockdown (June–September 2020), which included hand hygiene, reduction of social contacts and travel restrictions. Further, specific reasons (in predefined categories, with an open category for additional answers) for adherence (or not adhering) to Corona measures were elicited, and grouped to reflect more likely altruistic reasons, versus non-altruistic (i.e. self-centered) ones. The survey also elicited information regarding vaccine attitudes. Participants were asked how likely they would be to get vaccinated once a COVID-19 vaccine was available, need to independent scientific evaluation before vaccination; trust in Austrian government with regard to the safety of a COVID-19 vaccine, and dependence of vaccine acceptance on subsequent loosening of travel or other COVID restrictions.

### Statistical methods

Descriptive statistics were used to summarize the frequency and distribution of study population characteristics among Austrians, in the full sample and stratified by vaccine hesitancy. Summary statistics [mean, standard deviation (SD), or medians and interquartile ranges, and frequency (N and %)] were used to describe the characteristics or predictors of vaccine hesitancy in three groups (not hesitant, undecided and hesitant).

All analyses were conducted in the total population and stratified by gender. We present characteristics additionally stratified by voting behaviour during the last national election in Austria, which took place on 29 September 2019: voted for parties in power; opposition parties; or did not vote. In our primary analyses, we used age- and gender-adjusted (gender only in models using the total population), as well as multivariable adjusted multinomial logistic regression models to examine the association between various variables and vaccine hesitancy, and report OR and 95% CIs. In addition to age and gender, the multivariable models adjusted for the following covariables: area of residence (urban; rural ≥50.000 inhabitants; rural < 50.000 inhabitants); education (high school or less; Matura i.e. University entry exam, University degree); which party voted for during most recent national election (governing; opposition; did not vote); optimism scale, resilience scale and need for cognitive closure (each grouped into low or low-intermediate versus intermediate-high or high); main source to inform oneself about Corona (TV versus not using TV to inform oneself); subjective health status (very good; good; fair, bad or very bad).

Secondarily, we stratified by two groups of reasons for adherence (or non-adherence) to Corona measures, which we derived from a separate question that elicited reasons for adherence (or not adhering) to Corona measures. From all possible answers, we grouped as ‘altruistic reasons’ those participants who stated that *either* ‘protection of family’s health’ *or* ‘protection of the population’s health’ or ‘because they did not want to cause deaths’ were their primary reasons for adhering to COVID measures (*n* = 654) whereas those who listed none of these three reasons were grouped as ‘non-altruistic reasons’ (*n* = 271). A two-sided *p*-value of 0.05 or lower was considered statistically significant. All data analyses were performed using STATA (version 14.1, 2015, StataCorp LP).

## Notes

**Eva Schernhammer**, Professor

**Jakob Weitzer**, Research Assistant

**Manfred D. Laubichler**, Professor

**Brenda M. Birmann**, Assistant Professor

**Martin Bertau**, Professor

**Lukas Zenk**, Assistant Professor

**Guido Caniglia**, Professor

**Carlo C. Jäger**, Professor

**Gerald Steiner**, Professor
